# Bis(3-methyl-1-propyl-1*H*-imidazol-3-ium) bis­(4,6-disulfanidyl-4,6-disulfanyl­idene-1,2,3,5,4,6-tetra­thia­diphosphinane-κ^3^ *S* ^2^,*S* ^4^,*S* ^6^)nickel

**DOI:** 10.1107/S2414314620003120

**Published:** 2020-04-24

**Authors:** Lauren M. Dalecky, Christian A. Juillerat, Jason A. Cody

**Affiliations:** a Department of Chemistry, Lake Forest College, 555 N. Sheridan Rd., Lake Forest, IL 60045, USA; Dublin City University, Ireland

**Keywords:** crystal structure, ionothermal synthesis, imidazolium cation, thio­phosphate

## Abstract

The title salt, (PMIM)_2_[Ni(P_2_S_8_)_2_] (PMIM = 3-methyl-1-propyl-1*H*-imidazol-3-ium, C_7_H_13_N_2_^+^), consists of a nickel–thio­phosphate anion charge-balanced by a pair of crystallographically independent PMIM cations. It crystallizes in the monoclinic space group *P*2_1_/*n*. The structure exhibits the known [Ni(P_2_S_8_)_2_]^2−^ anion with two unique imidazolium cations in the asymmetric unit. Whereas one PMIM cation is well ordered, the other is disordered over two orientations with refined occupancies of 0.798 (2) and 0.202 (2). The salt was prepared directly from the elements in the ionic liquid [PMIM]CF_3_SO_3_. Whereas one of the PMIM cations is well behaved (it does not exhibit disorder even in the propyl side chain), the other is found in two overlapping positions. The refined occupancies for the two orientations are roughly 80:20. Here, too, there appears to be little disorder in the propyl arm.

## Abstract 2

The title salt, [PMIM]_2_[Ni(P_2_S_8_)_2_] (PMIM = 3-methyl-1-propyl-1*H*-imidazol-3-ium), was prepared from the elements in the ionic liquid [PMIM]CF_3_SO_3_. The structure consists of ordered anions and one ordered PMIM cation. The disordered PMIM cation is found in two orientations that refine to occupancies of roughly 0.80 and 0.20. The isolation of the title compound indicates that well behaved crystals can be obtained from direct reaction of the elements in ionic liquids with propyl chains that might otherwise be considered too prone to poor crystallization.

## Graphical abstract

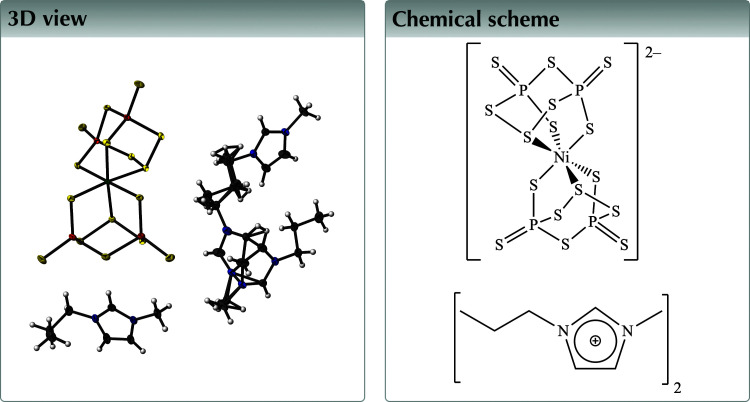

## Structure description

Ionothermal synthesis of inorganic compounds has received increased inter­est over the past two decades because of the high thermal stability, low vapor pressure, and reusability of ionic liquids (IL) (Wasserscheid & Welton, 2002 ▸; Freudenmann *et al.*, 2011 ▸; Zhang *et al.*, 2016 ▸). Ionothermal methods have been used to prepare a wide range of materials, including metal–organic frameworks (Cook *et al.*, 2013 ▸) and chalcogenides (Santner *et al.*, 2016 ▸).

Because of the inter­esting properties observed in metal thio­phosphates, especially luminescence (Huang *et al.*, 1992 ▸; Wu & Bensch, 2008 ▸), we have explored the preparation of these materials in ionic liquids. Ionothermal synthesis with nickel yielded four new nickel thio­phosphate anions: [Ni(P_2_S_8_)_2_]^2−^, [Ni(P_3_S_9_)(P_2_S_8_)]^3−^, [Ni(P_3_S_9_)_2_]^4−^, and [(NiP_3_S_8_)_4_(PS_4_)]^7−^, all crystallized with 1-ethyl-3-methyl­imidazolium [EMIM] cations from the IL (Cody *et al.*, 2012 ▸). The compound presented herein was synthesized by substitution of [EMIM] with 3-methyl-1-propyl­imidazolium [PMIM] cation, resulting in the most readily isolated anion of the group, [Ni(P_2_S_8_)_2_]^2−^, as a PMIM salt.

**Figure 1:**
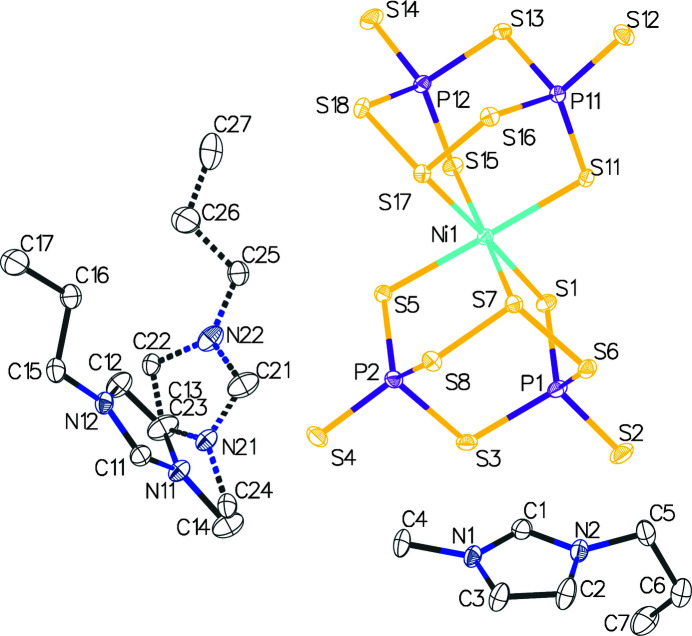
Structure of [PMIM]_2_[Ni(P_2_S_8_)_2_]. Displacement ellipsoids are drawn at the 50% probability level. Hydrogen atoms are omitted for clarity. The minor disorder component is shown with dashed bonds.

The structure consists of a single [Ni(P_2_S_8_)_2_]^2−^ anion (Cody *et al.*, 2012 ▸) and two PMIM cations. The anion exhibits the same shape as those previously isolated. The centrosymmetric space group *P*2_1_/*n* contains both optical isomers of the anion whereas Fig. 1 ▸ only shows the Δ isomer. Whereas one of the PMIM cations is well behaved (it does not exhibit disorder even in the propyl side chain), the other is found in two overlapping positions. The refined occupancies for the two orientations are roughly 80:20. Here, too, there appears to be little disorder in the propyl arm.

## Synthesis and crystallization

The ionic liquid 3-methyl-1-propyl-1*H*-imidazol-3-ium tri­fluoro­methane­sulfonate ([PMIM]CF_3_SO_3_) was prepared by a modified literature method (Bonhôte *et al.*, 1996 ▸): under a nitro­gen atmosphere, a stoichiometric amount of methyl tri­fluoro­methane­sulfonate was added dropwise to 1-propyl-1*H*-imidazole in di­chloro­methane.

Crystals of the title compound were prepared from a 125 mg mixture of the elements (ratio 1 Ni: 4 P: 16 S) that were weighed as a 1250 mg preparation, ground together, and portioned into Pyrex reaction tubes in a glove box. Then, in a glove bag, 1.25 ml portions of the ionic liquid [PMIM]CF_3_SO_3_ were added to the reaction tubes. The tubes were evacuated, sealed with a torch, heated at 150°C for 96 h, and then cooled to room temperature at a rate of 0.5°C /h. Similar crystals were obtained from a similar reaction in an ionic liquid with the same cation but different anion, [PMIM]BF_4_.

## Refinement

Crystal data, data collection and structure refinement details are summarized in Table 1 ▸.

The disorder of the PMIM cation was discovered by noticing slightly enlarged isotropic displacement parameters for the cation relative to the other cation in the structure. Also, residual electron density peaks near the cation formed a noticeable penta­gon, indicating the presence of the imidazolium core of the cation. The occupancies of the two disorder components refined to 0.798 (2) and 0.202 (2).

## Supplementary Material

Crystal structure: contains datablock(s) I. DOI: 10.1107/S2414314620003120/gg4003sup1.cif

Structure factors: contains datablock(s) I. DOI: 10.1107/S2414314620003120/gg4003Isup2.hkl

CCDC reference: 1988552

Additional supporting information:  crystallographic information; 3D view; checkCIF report

## Figures and Tables

**Table 1:** Experimental details

Crystal data	
Chemical formula	(C_7_H_13_N_2_)_2_[Ni(P_2_S_8_)_2_]
*M*_r_	945.94
Crystal system, space group	Monoclinic, *P*2_1_/*n*
Temperature (K)	100
*a*, *b*, *c* (Å)	23.042 (4), 7.1825 (12), 24.418 (4)
β (°)	117.505 (3)
*V* (Å^3^)	3584.3 (11)
*Z*	4
Radiation type	Mo *K*α
μ (mm^−1^)	1.67
Crystal size (mm)	0.28 × 0.16 × 0.05

Data collection	
Diffractometer	Bruker APEXII CCD
Absorption correction	Multi-scan (*SADABS*; Bruker, 2015 ▸)
*T*_min_, *T*_max_	0.655, 0.747
No. of measured, independent and observed [*I* > 2σ(*I*)] reflections	84130, 14239, 9766
*R*_int_	0.082
(sin θ/λ)_max_ (Å^−1^)	0.781

Refinement	
*R*[*F* ^2^ > 2σ(*F* ^2^)], *wR*(*F* ^2^), *S*	0.039, 0.083, 1.00
No. of reflections	14239
No. of parameters	388
No. of restraints	60
H-atom treatment	H-atom parameters constrained
Δρ_max_, Δρ_min_ (e Å^−3^)	0.96, −0.52

Computer programs: *APEX2* and *SAINT* (Bruker, 2015 ▸), *SHELXS* (Sheldrick, 2008 ▸), *SHELXL2014* (Sheldrick, 2015 ▸) and *OLEX2* (Dolomanov *et al.*, 2009 ▸).

## full crystallographic data

### Crystal data

**Table:**

(C_7_H_13_N_2_)_2_[Ni(P_2_S_8_)_2_]	*F*(000) = 1928
*M**_r_* = 945.94	*D*_x_ = 1.753 Mg m^−^^3^
Monoclinic, *P*2_1_/*n*	Mo *K*α radiation, λ = 0.71073 Å
*a* = 23.042 (4) Å	Cell parameters from 9334 reflections
*b* = 7.1825 (12) Å	θ = 2.5–31.7°
*c* = 24.418 (4) Å	µ = 1.67 mm^−^^1^
β = 117.505 (3)°	*T* = 100 K
*V* = 3584.3 (11) Å^3^	Needle, dark orange
*Z* = 4	0.28 × 0.16 × 0.05 mm

### Data collection

**Table:**

Bruker APEXII CCD diffractometer	9766 reflections with *I* > 2σ(*I*)
Graphite monochromator	*R*_int_ = 0.082
φ and ω scans	θ_max_ = 33.7°, θ_min_ = 2.5°
Absorption correction: multi-scan (SADABS; Bruker, 2015)	*h* = −35→35
*T*_min_ = 0.655, *T*_max_ = 0.747	*k* = −10→11
84130 measured reflections	*l* = −38→37
14239 independent reflections	

### Refinement

**Table:**

Refinement on *F*^2^	Primary atom site location: structure-invariant direct methods
Least-squares matrix: full	Hydrogen site location: inferred from neighbouring sites
*R*[*F*^2^ > 2σ(*F*^2^)] = 0.039	H-atom parameters constrained
*wR*(*F*^2^) = 0.083	*w* = 1/[σ^2^(*F*_o_^2^) + (0.0312*P*)^2^ + 1.1383*P*] where *P* = (*F*_o_^2^ + 2*F*_c_^2^)/3
*S* = 1.00	(Δ/σ)_max_ = 0.002
14239 reflections	Δρ_max_ = 0.96 e Å^−^^3^
388 parameters	Δρ_min_ = −0.52 e Å^−^^3^
60 restraints	

### Special details

**Table:**

Geometry. All esds (except the esd in the dihedral angle between two l.s. planes) are estimated using the full covariance matrix. The cell esds are taken into account individually in the estimation of esds in distances, angles and torsion angles; correlations between esds in cell parameters are only used when they are defined by crystal symmetry. An approximate (isotropic) treatment of cell esds is used for estimating esds involving l.s. planes.
Refinement. All H atoms were positions with idealized geometry (methyl H atoms allowed to rotate but not to tip) and were refined isotropic with *U*_iso_(H) = 1.2 *U*_eq_(C) (1.5 for methyl H atoms) using a riding model with C—H = 0.93 Å for aromatic, 0.97 Å for methylene and 0.96 Å for methyl H-atoms.

### Fractional atomic coordinates and isotropic or equivalent isotropic displacement parameters (Å^2^)

**Table:**

	*x*	*y*	*z*	*U*_iso_*/*U*_eq_	Occ. (<1)
Ni1	0.52291 (2)	0.16445 (4)	0.27543 (2)	0.01107 (6)	
S1	0.58517 (3)	−0.10825 (7)	0.28600 (2)	0.01437 (10)	
S2	0.74680 (3)	−0.17840 (9)	0.33188 (3)	0.02335 (13)	
S3	0.70711 (3)	0.06248 (8)	0.42211 (2)	0.01638 (11)	
S4	0.67644 (3)	0.33269 (9)	0.51111 (2)	0.02210 (12)	
S5	0.54721 (3)	0.16876 (8)	0.38156 (2)	0.01444 (10)	
S6	0.68504 (3)	0.23021 (8)	0.28616 (2)	0.01543 (10)	
S7	0.61254 (3)	0.38979 (7)	0.28950 (2)	0.01320 (10)	
S8	0.65016 (3)	0.49267 (7)	0.37821 (2)	0.01545 (10)	
S11	0.49662 (3)	0.16109 (8)	0.16862 (2)	0.01392 (10)	
S12	0.38843 (3)	0.40281 (8)	0.04311 (2)	0.01923 (11)	
S13	0.33544 (2)	0.22267 (8)	0.13143 (2)	0.01423 (10)	
S14	0.27374 (3)	0.07818 (9)	0.21939 (3)	0.02258 (12)	
S15	0.43218 (3)	−0.02240 (7)	0.26043 (2)	0.01456 (10)	
S16	0.43958 (3)	0.58100 (7)	0.18019 (2)	0.01442 (10)	
S17	0.46596 (3)	0.47461 (7)	0.26699 (2)	0.01354 (10)	
S18	0.38028 (3)	0.41072 (8)	0.26949 (2)	0.01621 (10)	
P1	0.67724 (3)	−0.01963 (8)	0.32921 (3)	0.01460 (11)	
P2	0.63910 (3)	0.25869 (8)	0.42497 (2)	0.01368 (11)	
P11	0.42011 (3)	0.33097 (8)	0.12893 (2)	0.01227 (10)	
P12	0.35650 (3)	0.15073 (8)	0.22274 (3)	0.01355 (10)	
N1	0.85499 (9)	0.5648 (3)	0.47085 (8)	0.0168 (4)	
N2	0.87391 (9)	0.4589 (3)	0.39798 (9)	0.0180 (4)	
C1	0.83490 (11)	0.4454 (3)	0.42436 (10)	0.0191 (4)	
H1	0.7986	0.3636	0.4119	0.023*	
C2	0.92065 (13)	0.5906 (3)	0.42907 (12)	0.0265 (5)	
H2	0.9550	0.6280	0.4203	0.032*	
C3	0.90864 (12)	0.6573 (3)	0.47467 (12)	0.0237 (5)	
H3	0.9329	0.7508	0.5038	0.028*	
C4	0.82347 (12)	0.5986 (4)	0.50984 (11)	0.0237 (5)	
H4A	0.8004	0.7182	0.4986	0.036*	
H4B	0.8567	0.6019	0.5532	0.036*	
H4C	0.7922	0.4986	0.5038	0.036*	
C5	0.86870 (13)	0.3488 (4)	0.34505 (11)	0.0262 (5)	
H5A	0.8669	0.4339	0.3124	0.031*	
H5B	0.8276	0.2760	0.3278	0.031*	
C6	0.92698 (13)	0.2153 (4)	0.36383 (12)	0.0277 (5)	
H6A	0.9222	0.1468	0.3268	0.033*	
H6B	0.9678	0.2892	0.3795	0.033*	
C7	0.93295 (14)	0.0783 (4)	0.41199 (14)	0.0344 (6)	
H7A	0.8916	0.0105	0.3980	0.052*	
H7B	0.9429	0.1442	0.4505	0.052*	
H7C	0.9682	−0.0098	0.4191	0.052*	
N11	0.70511 (12)	0.8449 (3)	0.60795 (12)	0.0189 (5)	0.798 (2)
N12	0.63038 (12)	1.0204 (3)	0.60984 (11)	0.0169 (5)	0.798 (2)
C11	0.69112 (14)	1.0146 (4)	0.61822 (13)	0.0182 (6)	0.798 (2)
H11	0.7203	1.1174	0.6299	0.022*	0.798 (2)
C12	0.60435 (15)	0.8442 (4)	0.59479 (15)	0.0245 (6)	0.798 (2)
H12	0.5613	0.8066	0.5856	0.029*	0.798 (2)
C13	0.65107 (13)	0.7397 (4)	0.59582 (13)	0.0317 (6)	0.798 (2)
H13	0.6480	0.6089	0.5891	0.038*	0.798 (2)
C14	0.76729 (16)	0.7843 (5)	0.61090 (19)	0.0282 (8)	0.798 (2)
H14A	0.7804	0.8720	0.5878	0.042*	0.798 (2)
H14B	0.7620	0.6599	0.5927	0.042*	0.798 (2)
H14C	0.8011	0.7806	0.6541	0.042*	0.798 (2)
C15	0.5966 (2)	1.1859 (6)	0.6159 (2)	0.0209 (8)	0.798 (2)
H15A	0.6255	1.2956	0.6244	0.025*	0.798 (2)
H15B	0.5872	1.1693	0.6513	0.025*	0.798 (2)
C16	0.5327 (2)	1.2216 (10)	0.5576 (2)	0.0222 (7)	0.798 (2)
H16A	0.5423	1.2574	0.5235	0.027*	0.798 (2)
H16B	0.5062	1.1063	0.5455	0.027*	0.798 (2)
C17	0.4944 (2)	1.3755 (6)	0.56866 (18)	0.0422 (10)	0.798 (2)
H17A	0.4555	1.4046	0.5299	0.063*	0.798 (2)
H17B	0.5219	1.4868	0.5837	0.063*	0.798 (2)
H17C	0.4812	1.3347	0.5995	0.063*	0.798 (2)
N21	0.6813 (4)	0.8140 (13)	0.5709 (4)	0.0189 (5)	0.202 (2)
N22	0.5807 (4)	0.7520 (14)	0.5062 (4)	0.0245 (6)	0.202 (2)
C21	0.6387 (5)	0.7999 (18)	0.5113 (5)	0.0317 (6)	0.202 (2)
H21	0.6482	0.8208	0.4778	0.038*	0.202 (2)
C22	0.5866 (4)	0.7297 (15)	0.5644 (4)	0.0169 (5)	0.202 (2)
H22	0.5544	0.7124	0.5778	0.020*	0.202 (2)
C23	0.65107 (13)	0.7397 (4)	0.59582 (13)	0.0317 (6)	0.202 (2)
H23	0.6736	0.6910	0.6365	0.038*	0.202 (2)
C24	0.7522 (5)	0.8455 (19)	0.5975 (6)	0.0209 (8)	0.202 (2)
H24A	0.7620	0.9073	0.5669	0.031*	0.202 (2)
H24B	0.7667	0.9244	0.6342	0.031*	0.202 (2)
H24C	0.7751	0.7257	0.6090	0.031*	0.202 (2)
C25	0.5235 (5)	0.7101 (18)	0.4475 (5)	0.0222 (7)	0.202 (2)
H25A	0.5190	0.5736	0.4415	0.027*	0.202 (2)
H25B	0.5295	0.7644	0.4132	0.027*	0.202 (2)
C26	0.4624 (9)	0.788 (6)	0.4465 (13)	0.0422 (10)	0.202 (2)
H26A	0.4675	0.9248	0.4530	0.051*	0.202 (2)
H26B	0.4571	0.7343	0.4812	0.051*	0.202 (2)
C27	0.4014 (10)	0.750 (2)	0.3869 (10)	0.032 (5)	0.202 (2)
H27A	0.4036	0.8177	0.3531	0.048*	0.202 (2)
H27B	0.3629	0.7912	0.3908	0.048*	0.202 (2)
H27C	0.3980	0.6161	0.3782	0.048*	0.202 (2)

### Atomic displacement parameters (Å^2^)

**Table:**

	*U*^11^	*U*^22^	*U*^33^	*U*^12^	*U*^13^	*U*^23^
Ni1	0.00997 (12)	0.01233 (12)	0.01185 (12)	0.00070 (10)	0.00585 (10)	0.00124 (9)
S1	0.0126 (2)	0.0121 (2)	0.0182 (2)	0.00127 (18)	0.0069 (2)	0.00146 (19)
S2	0.0171 (3)	0.0269 (3)	0.0281 (3)	0.0101 (2)	0.0121 (2)	0.0059 (2)
S3	0.0119 (2)	0.0202 (3)	0.0146 (2)	0.0027 (2)	0.0040 (2)	0.00467 (19)
S4	0.0189 (3)	0.0338 (3)	0.0114 (2)	−0.0049 (2)	0.0052 (2)	−0.0013 (2)
S5	0.0114 (2)	0.0203 (3)	0.0125 (2)	−0.00157 (19)	0.00631 (19)	0.00121 (19)
S6	0.0133 (2)	0.0187 (3)	0.0167 (2)	0.0002 (2)	0.0089 (2)	0.00277 (19)
S7	0.0132 (2)	0.0142 (2)	0.0127 (2)	0.00025 (18)	0.00633 (19)	0.00244 (18)
S8	0.0171 (3)	0.0149 (2)	0.0141 (2)	−0.0028 (2)	0.0069 (2)	0.00007 (19)
S11	0.0133 (2)	0.0174 (2)	0.0131 (2)	0.00321 (19)	0.00789 (19)	0.00051 (19)
S12	0.0207 (3)	0.0240 (3)	0.0121 (2)	−0.0008 (2)	0.0068 (2)	0.0036 (2)
S13	0.0102 (2)	0.0184 (3)	0.0131 (2)	−0.00093 (19)	0.00459 (19)	−0.00028 (18)
S14	0.0127 (3)	0.0314 (3)	0.0267 (3)	−0.0028 (2)	0.0117 (2)	0.0037 (2)
S15	0.0129 (2)	0.0133 (2)	0.0179 (2)	−0.00071 (19)	0.0075 (2)	0.00236 (19)
S16	0.0156 (2)	0.0126 (2)	0.0151 (2)	0.00023 (19)	0.0071 (2)	0.00093 (18)
S17	0.0135 (2)	0.0141 (2)	0.0131 (2)	0.00046 (19)	0.00628 (19)	−0.00095 (18)
S18	0.0154 (3)	0.0196 (3)	0.0175 (2)	0.0016 (2)	0.0109 (2)	−0.0018 (2)
P1	0.0113 (2)	0.0162 (3)	0.0168 (3)	0.0028 (2)	0.0069 (2)	0.0037 (2)
P2	0.0122 (3)	0.0170 (3)	0.0116 (2)	−0.0007 (2)	0.0053 (2)	0.00183 (19)
P11	0.0118 (2)	0.0146 (3)	0.0109 (2)	0.0006 (2)	0.0057 (2)	0.00054 (19)
P12	0.0106 (2)	0.0167 (3)	0.0147 (2)	−0.0007 (2)	0.0070 (2)	0.0006 (2)
N1	0.0181 (9)	0.0189 (9)	0.0184 (9)	0.0016 (7)	0.0126 (8)	0.0030 (7)
N2	0.0208 (10)	0.0189 (9)	0.0188 (9)	0.0028 (7)	0.0129 (8)	0.0015 (7)
C1	0.0169 (11)	0.0234 (12)	0.0182 (10)	0.0008 (9)	0.0091 (9)	0.0025 (9)
C2	0.0344 (14)	0.0203 (12)	0.0395 (14)	−0.0050 (10)	0.0295 (13)	−0.0042 (10)
C3	0.0268 (13)	0.0194 (11)	0.0346 (13)	−0.0051 (10)	0.0226 (11)	−0.0062 (10)
C4	0.0295 (13)	0.0267 (13)	0.0260 (12)	0.0015 (10)	0.0222 (11)	0.0008 (10)
C5	0.0309 (14)	0.0334 (14)	0.0173 (11)	0.0042 (11)	0.0135 (10)	−0.0007 (10)
C6	0.0317 (14)	0.0282 (13)	0.0307 (13)	0.0006 (11)	0.0208 (12)	−0.0044 (10)
C7	0.0338 (15)	0.0298 (15)	0.0444 (17)	0.0094 (12)	0.0222 (14)	0.0048 (12)
N11	0.0159 (12)	0.0191 (11)	0.0248 (12)	0.0003 (9)	0.0120 (10)	0.0012 (10)
N12	0.0197 (12)	0.0170 (11)	0.0175 (11)	0.0006 (9)	0.0116 (9)	0.0016 (8)
C11	0.0168 (13)	0.0191 (14)	0.0199 (13)	−0.0024 (11)	0.0095 (11)	−0.0001 (10)
C12	0.0207 (14)	0.0201 (14)	0.0375 (17)	−0.0034 (11)	0.0176 (13)	−0.0002 (12)
C13	0.0223 (13)	0.0242 (13)	0.0455 (16)	−0.0045 (10)	0.0128 (12)	0.0054 (11)
C14	0.0221 (17)	0.0242 (17)	0.045 (2)	0.0051 (13)	0.0210 (16)	0.0031 (15)
C15	0.0254 (18)	0.021 (2)	0.0199 (16)	0.0015 (16)	0.0134 (13)	0.0003 (17)
C16	0.0253 (16)	0.0199 (17)	0.0224 (16)	−0.0002 (13)	0.0120 (12)	−0.0034 (12)
C17	0.040 (2)	0.040 (2)	0.034 (2)	0.0199 (17)	0.0063 (17)	−0.0089 (16)
N21	0.0159 (12)	0.0191 (11)	0.0248 (12)	0.0003 (9)	0.0120 (10)	0.0012 (10)
N22	0.0207 (14)	0.0201 (14)	0.0375 (17)	−0.0034 (11)	0.0176 (13)	−0.0002 (12)
C21	0.0223 (13)	0.0242 (13)	0.0455 (16)	−0.0045 (10)	0.0128 (12)	0.0054 (11)
C22	0.0197 (12)	0.0170 (11)	0.0175 (11)	0.0006 (9)	0.0116 (9)	0.0016 (8)
C23	0.0223 (13)	0.0242 (13)	0.0455 (16)	−0.0045 (10)	0.0128 (12)	0.0054 (11)
C24	0.0254 (18)	0.021 (2)	0.0199 (16)	0.0015 (16)	0.0134 (13)	0.0003 (17)
C25	0.0253 (16)	0.0199 (17)	0.0224 (16)	−0.0002 (13)	0.0120 (12)	−0.0034 (12)
C26	0.040 (2)	0.040 (2)	0.034 (2)	0.0199 (17)	0.0063 (17)	−0.0089 (16)
C27	0.052 (11)	0.020 (9)	0.040 (9)	−0.002 (9)	0.036 (8)	0.003 (8)

### Geometric parameters (Å, º)

**Table:**

Ni1—S1	2.3705 (7)	C2—C3	1.353 (3)
Ni1—S5	2.3852 (7)	C5—C6	1.538 (4)
Ni1—S7	2.5195 (7)	C6—C7	1.490 (4)
Ni1—S11	2.3897 (7)	N11—C11	1.314 (4)
Ni1—S15	2.3662 (7)	N11—C13	1.367 (3)
Ni1—S17	2.5450 (7)	N11—C14	1.467 (4)
S1—P1	1.9879 (8)	N11—N21	0.843 (9)
S2—P1	1.9436 (8)	N11—C23	1.367 (3)
S3—P1	2.1272 (9)	N11—C24	1.224 (12)
S3—P2	2.1325 (8)	N12—C11	1.318 (3)
S4—P2	1.9431 (8)	N12—C12	1.375 (4)
S5—P2	1.9877 (8)	N12—C15	1.465 (5)
S6—S7	2.0583 (8)	C11—N21	1.795 (10)
S6—P1	2.1290 (8)	C12—C13	1.303 (4)
S7—S8	2.0629 (8)	C12—C22	1.056 (11)
S8—P2	2.1123 (8)	C12—C23	1.303 (4)
S11—P11	1.9893 (8)	C13—N21	1.238 (8)
S12—P11	1.9435 (8)	C13—C22	1.321 (9)
S13—P11	2.1273 (8)	C14—N21	1.771 (9)
S13—P12	2.1152 (8)	C14—C24	0.562 (12)
S14—P12	1.9419 (8)	C15—C16	1.523 (6)
S15—P12	1.9879 (8)	C16—C17	1.514 (6)
S16—S17	2.0629 (8)	N21—C21	1.332 (12)
S16—P11	2.1153 (8)	N21—C23	1.238 (8)
S17—S18	2.0552 (8)	N21—C24	1.469 (11)
S18—P12	2.1243 (8)	N22—C21	1.329 (11)
N1—C1	1.324 (3)	N22—C22	1.371 (11)
N1—C3	1.368 (3)	N22—C25	1.462 (11)
N1—C4	1.460 (3)	C22—C23	1.321 (9)
N2—C1	1.329 (3)	C25—C26	1.508 (16)
N2—C2	1.369 (3)	C26—C27	1.510 (16)
N2—C5	1.472 (3)		
			
S1—Ni1—S5	93.78 (2)	C11—N11—C14	125.9 (3)
S1—Ni1—S7	95.69 (2)	C11—N11—C23	106.0 (2)
S1—Ni1—S11	86.91 (2)	C13—N11—C14	128.1 (3)
S1—Ni1—S17	174.59 (2)	N21—N11—C11	110.8 (7)
S5—Ni1—S7	94.43 (2)	N21—N11—C13	63.0 (6)
S5—Ni1—S11	179.01 (2)	N21—N11—C14	96.3 (7)
S5—Ni1—S17	86.00 (2)	N21—N11—C23	63.0 (6)
S7—Ni1—S17	78.95 (2)	N21—N11—C24	88.6 (8)
S11—Ni1—S7	86.21 (2)	C23—N11—C14	128.1 (3)
S11—Ni1—S17	93.387 (19)	C24—N11—C11	110.9 (7)
S15—Ni1—S1	89.73 (2)	C24—N11—C13	139.7 (7)
S15—Ni1—S5	85.57 (2)	C24—N11—C23	139.7 (7)
S15—Ni1—S7	174.57 (2)	C11—N12—C12	108.2 (2)
S15—Ni1—S11	93.72 (2)	C11—N12—C15	125.8 (3)
S15—Ni1—S17	95.64 (2)	C12—N12—C15	125.9 (3)
P1—S1—Ni1	103.71 (3)	N11—C11—N12	109.6 (2)
P1—S3—P2	109.72 (3)	N12—C11—N21	96.7 (3)
P2—S5—Ni1	103.97 (3)	C13—C12—N12	105.7 (3)
S7—S6—P1	101.22 (3)	C22—C12—N12	152.1 (6)
S6—S7—Ni1	105.43 (3)	C22—C12—C13	67.2 (5)
S6—S7—S8	106.62 (3)	C22—C12—C23	67.2 (5)
S8—S7—Ni1	107.08 (3)	C23—C12—N12	105.7 (3)
S7—S8—P2	100.69 (3)	C12—C13—N11	110.3 (3)
P11—S11—Ni1	104.27 (3)	C12—C13—C22	47.5 (5)
P12—S13—P11	110.65 (3)	N21—C13—C12	112.9 (5)
P12—S15—Ni1	103.90 (3)	N21—C13—C22	118.3 (6)
S17—S16—P11	100.07 (3)	C22—C13—N11	145.3 (5)
S16—S17—Ni1	107.68 (3)	C24—C14—N11	54.0 (13)
S18—S17—Ni1	105.58 (3)	C24—C14—N21	49.5 (13)
S18—S17—S16	106.38 (3)	N12—C15—C16	111.9 (4)
S17—S18—P12	101.14 (3)	C17—C16—C15	110.4 (4)
S1—P1—S3	113.28 (3)	N11—N21—C13	79.7 (7)
S1—P1—S6	108.70 (3)	N11—N21—C14	55.4 (5)
S2—P1—S1	119.43 (4)	N11—N21—C21	168.3 (13)
S2—P1—S3	105.74 (3)	N11—N21—C23	79.7 (7)
S2—P1—S6	104.75 (4)	N11—N21—C24	56.4 (7)
S3—P1—S6	103.49 (3)	C13—N21—C11	87.8 (5)
S4—P2—S3	104.56 (3)	C13—N21—C14	114.7 (6)
S4—P2—S5	119.76 (4)	C13—N21—C21	101.9 (8)
S4—P2—S8	105.29 (4)	C13—N21—C24	127.8 (9)
S5—P2—S3	112.69 (4)	C14—N21—C11	88.0 (4)
S5—P2—S8	109.57 (3)	C21—N21—C11	125.1 (9)
S8—P2—S3	103.54 (3)	C21—N21—C14	131.7 (8)
S11—P11—S13	112.33 (3)	C21—N21—C24	127.0 (9)
S11—P11—S16	109.54 (3)	C23—N21—C11	87.8 (5)
S12—P11—S11	119.68 (4)	C23—N21—C14	114.7 (6)
S12—P11—S13	103.60 (3)	C23—N21—C21	101.9 (8)
S12—P11—S16	106.50 (3)	C23—N21—C24	127.8 (9)
S16—P11—S13	103.89 (3)	C24—N21—C11	79.0 (7)
S13—P12—S18	103.20 (3)	C21—N22—C22	108.6 (8)
S14—P12—S13	106.16 (3)	C21—N22—C25	123.8 (9)
S14—P12—S15	119.08 (4)	C22—N22—C25	127.2 (9)
S14—P12—S18	105.25 (3)	N22—C21—N21	108.8 (10)
S15—P12—S13	111.47 (3)	C12—C22—C13	65.4 (5)
S15—P12—S18	110.35 (3)	C12—C22—N22	117.3 (10)
C1—N1—C3	108.68 (19)	C12—C22—C23	65.4 (5)
C1—N1—C4	125.8 (2)	C13—C22—N22	98.2 (7)
C3—N1—C4	125.5 (2)	C23—C22—N22	98.2 (7)
C1—N2—C2	108.46 (19)	C12—C23—N11	110.3 (3)
C1—N2—C5	125.9 (2)	C12—C23—C22	47.5 (5)
C2—N2—C5	125.6 (2)	N21—C23—C12	112.9 (5)
N1—C1—N2	108.7 (2)	N21—C23—C22	118.3 (6)
C3—C2—N2	107.1 (2)	C22—C23—N11	145.3 (5)
C2—C3—N1	107.1 (2)	C14—C24—N11	104.2 (17)
N2—C5—C6	111.6 (2)	C14—C24—N21	113.6 (17)
C7—C6—C5	113.4 (2)	N22—C25—C26	110.5 (12)
C11—N11—C13	106.0 (2)	C25—C26—C27	113.2 (17)
